# Gabapentin and pregabalin exposures reported to United States poison centers, 2012–2022

**DOI:** 10.1186/s40621-024-00547-9

**Published:** 2024-11-01

**Authors:** Emily J.R. Carter, Natalie I. Rine, Sandhya Kistamgari, Hannah L. Hays, Henry A. Spiller, Jingzhen Yang, Motao Zhu, Gary A. Smith

**Affiliations:** 1 Center for Injury Research and Policy, The Abigail Wexner Research Institute at Nationwide Children’s Hospital, 700 Children’s Drive, Columbus, OH 43205 USA; 2 Kirk Kerkorian School of Medicine, Las Vegas, NV USA; 3 Central Ohio Poison Center, Nationwide Children’s Hospital, Columbus, OH USA; 4 Department of Pediatrics, The Ohio State University College of Medicine, Columbus, OH USA; 5 Child Injury Prevention Alliance, Columbus, OH USA

**Keywords:** Gabapentin, Pregabalin, Gabapentinoid, Toxicity, Suicide

## Abstract

**Background:**

Gabapentin and pregabalin were originally introduced as anticonvulsant medications but are now also prescribed on- and off-label for multiple medical disorders, especially for pain management. The national opioid crisis has led to increased use of non-opioid pain medications, including gabapentinoids, which has been associated with changing patterns of adverse events associated with these medications. This study investigated the characteristics and trends of gabapentin and pregabalin exposures reported to US poison centers from 2012 to 2022.

**Methods:**

National Poison Data System data involving gabapentin and pregabalin exposures for 2012 to 2022 were analyzed.

**Results:**

There were 124,161 exposures involving gabapentin and pregabalin as the primary substance reported to US poison centers during the study period. Most exposures involved gabapentin (85.9%), females (59.4%), single-substance exposures (62.9%), or occurred at a residence (97.2%). Suspected suicides accounted for 45.2% of exposures. Most exposures were associated with a minor effect (27.4%) or no effect (34.0%), while 22.1% experienced a serious medical outcome, including 96 fatalities. The rate of gabapentin and pregabalin exposures per one million US population increased by 236.1% from 22.7 in 2012 to 76.5 in 2019 (*P* < 0.001), followed by a non-significant decrease to 68.5 in 2022 (*P* = 0.068).

**Conclusions:**

The rate of gabapentin and pregabalin exposures reported to US poison centers increased by more than 230% from 2012 to 2019 before plateauing from 2019 to 2022. The observed rate trend was driven primarily by gabapentin exposures and by cases associated with suspected suicide. Although most exposures were associated with a minor or no effect, 22% of individuals experienced a serious medical outcome, including 96 fatalities. These findings contribute to the discussion of rescheduling gabapentin as a federally controlled substance, which is the current status of pregabalin. Prevention of suicide associated with gabapentin and pregabalin merits special attention.

## Background

Gabapentin and pregabalin are gamma-aminobutyric acid analogs that were originally introduced as anticonvulsant medications. They are also prescribed for post-herpetic neuralgia, fibromyalgia, and neuropathic pain (Faryar et al. 2019; Klein-Schwartz et al. 2003; Reynolds et al. 2020; Tharp et al. 2019; Pfizer 2017, 2018). In addition, gabapentin is used off-label for mood disorders, mild alcohol withdrawal, alcohol use disorder, migraine headache, chronic pain, and post-operative pain management (Klein-Schwartz et al. 2003; Reynolds et al. 2020; Ghai et al. 2011; Miller and Price 2009; Sirven 2010). Gabapentin is the most commonly prescribed of the gabapentinoids and was the seventh most commonly prescribed medication in the United States (US) in 2019 (Mattson et al. 2022). The crisis of opioid misuse nationally has led to increased use of non-opioid medications, including gabapentinoids, for pain management (Goins et al. 2021). Unlike pregabalin, gabapentin is not a federally controlled substance under the Controlled Substances Act; however, it is a scheduled substance in at least seven states (Peckham et al. 2018; Sharkey 2023; US Drug Enforcement Administration 2023).

Adverse effects of gabapentin include dizziness, ataxia, tremors, and respiratory depression (Faryar et al. 2019; Pfizer 2017; Wills et al.  2014). Likewise, adverse effects of pregabalin include dizziness, somnolence and sedation (Pfizer 2018; Ghai et al. 2011). Co-use of gabapentin or pregabalin with other substances, including opioids, is common and may potentiate effects, such as euphoria and respiratory depression (Faryar et al. 2019; Mattson et al. 2022; Peckham et al. 2018; US Food and Drug Administration 2020). Based on this concern, the US Food and Drug Administration (FDA) issued a drug safety communication in December 2019, which required manufacturers to add warnings about respiratory depression to the prescribing information for gabapentinoids (US Food and Drug Administration 2020).

Previous studies on gabapentin and pregabalin have had limitations in their scope, including some focusing on only regional data (Faryar et al. 2019; Klein-Schwartz et al. 2003; Wills et al. 2014), specific age groups (Faryar et al. 2019; Reynolds et al. 2020; Wills et al. 2014; Toce et al. 2022), or fatalities (Tharp et al. 2019; Greene et al. 2023). The most recent study to publish gabapentin-related reporting trends to poison centers (PCs) nationally included data through 2017 (Reynolds et al. 2020). The objective of this study is to update our knowledge about the characteristics and trends of exposures involving gabapentin and pregabalin reported to US PCs from 2012 to 2022.

## Methods

### Data sources

Deidentified study data were obtained from the National Poison Data System (NPDS), which is a database maintained by America’s Poison Centers (America’s Poison Centers 2023). The database collects information in near real-time from calls to PCs in the US and its territories. Inter-censal and post-censal population estimates obtained from the US Census Bureau from 2012 to 2022 were used to calculate population-based exposure rates, including age-specific and sex-specific rates (US Census Bureau  2010, 2020). This study was determined to be exempt from approval by the institutional review board of the authors’ institution.

## Case selection

Data for cases involving exposures to gabapentin and pregabalin (based on NPDS generic codes) reported to US PCs from January 1, 2012, through December 31, 2022, were obtained from the NPDS. Cases were excluded from the study if (1) the medical outcome was coded as “confirmed non-exposure” (*n* = 1,290) or “unrelated effect” (*n* = 14,519) or (2) the reason for exposure was coded as “adverse reaction-food” (*n* = 43), “unintentional-bite/sting” (*n* = 24), or “unintentional-food poisoning” (*n* = 42). In addition, fatal cases were excluded from the study if the relative contribution to fatality for gabapentin or pregabalin was determined by America’s Poison Centers to be “clearly not responsible” or “probably not responsible” (*n* = 14). This yielded a total of 228,233 exposure cases.

## Study variables

Age groups were categorized as (1) ≤ 5 years, (2) 6–19 years, (3) 20–29 years, (4) 30–39 years, (5) 40–49 years, (6) 50–59 years, and (7) ≥ 60 years. Based on NPDS categories, the reason for exposure was grouped as (1) unintentional-general, (2) unintentional-therapeutic error, (3) unintentional-other (includes unintentional-environmental, unintentional-occupational, and unintentional-misuse), (4) unintentional-unknown, (5) intentional-suspected suicide, (6) intentional-misuse, (7) intentional-abuse, (8) intentional-unknown, and (9) other (includes adverse reaction-drug, adverse reaction-other, other-contamination/tampering, other-malicious, and other-withdrawal). Exposure sites were grouped as (1) residence (includes own residence and other residence), (2) other (includes school, healthcare facility, workplace, public area, and restaurant/food service), and (3) unknown.

Based on NPDS categories, the highest level of health care received was grouped as (1) no healthcare facility treatment received (includes the NPDS categories “managed on site” [not in a healthcare facility] and “other” management site), (2) treated/evaluated and released, (3) admitted to a critical care unit (CCU), (4) admitted to a non-CCU, (5) admitted to a psychiatric facility, (6) refused referral/did not arrive at healthcare facility, and (7) unknown (includes patient lost to follow-up and left against medical advice). For the purpose of analysis, admitted to a CCU and admitted to a non-CCU were combined into medical admission.

Based on NPDS categories, medical outcome was grouped as (1) no effect, (2) minor effect (minimally bothersome symptoms that generally resolve rapidly with no residual disability), (3) moderate effect (more pronounced, prolonged, or systemic than minor symptoms), (4) major effect (symptoms are life-threatening or result in significant disability or disfigurement), (5) death, (6) not followed (includes minimal clinical effects possible and judged as non-toxic exposure), and (7) unknown (includes unable to follow [potentially toxic exposure]). For the purpose of analysis, moderate effect, major effect, and death were combined into “serious medical outcome.” Additional variables included substance category (gabapentin or pregabalin), sex, year, and exposure type (single-substance or multiple-substance).

### Statistical analysis

Data were analyzed using IBM Statistics SPSS 28 for Windows (IBM Corp, Armonk, NY) and SAS 9.4 (SAS Institute Inc, Cary, NC) statistical software. Exposures involving gabapentin or pregabalin as the primary substance (including both single-substance and multiple-substance exposures) were used to analyze the general characteristics of gabapentin and pregabalin exposures. A primary substance is the substance most likely responsible for the observed clinical effects as determined by the specialists in poison information at a PC. Trends over time were determined using all reported exposures (including primary and non-primary substance exposures). Piecewise and simple linear regression models were used, as appropriate, to determine the statistical significance of trends by evaluating whether the null hypothesis of slope = 0 could be rejected. Break points for piecewise analyses were determined based on scatter plots. Statistical significance was determined at α = 0.05. Other analyses included the calculation of risk ratios (RRs) with 95% confidence intervals (CIs).

## Results

### General characteristics

There were 124,161 exposures for which gabapentin or pregabalin was the primary substance. Most involved females (59.4%), were single-substance exposures (62.9%), occurred at a residence (97.2%), or were already in or en route to a healthcare facility when the PC was contacted (Table 1). Gabapentin accounted for 85.9% and pregabalin accounted for 14.1% of these exposures (Table 2).

**Table 1:** Characteristics of exposures involving gabapentin or pregabalin as the primary substance reported to the national poison data system by age group, 2012–2022

Characteristics	Age group								
	≤ 5 years *n* (%)^a^	6–19 years *n* (%)^a^	20–29 years *n* (%)^a^	30–39 years *n* (%)^a^	40–49 years *n* (%)^a^	50–59 years *n* (%)^a^	≥ 60 years *n* (%)^a^	Unknown	Total *n* (%)^a^
**Sex**									
Male	7,935 (51.1)	3,747 (36.9)	6,860 (44.0)	8,332 (42.2)	7,246 (38.1)	7,004 (38.7)	7,293 (35.3)	1,849	50,266 (40.6)
Female	7,599 (48.9)	6,398 (63.1)	8,722 (56.0)	11,403 (57.8)	11,762 (61.9)	11,094 (61.3)	13,375 (64.7)	3,098	73,451 (59.4)
Unknown	45	11	16	16	14	8	20	314	444
**Type of exposure**									
Single-substance	13,498 (86.6)	6,851 (67.5)	8,838 (56.7)	10,733 (54.3)	10,052 (52.8)	10,085 (55.7)	14,049 (67.9)	4,122	78,228 (62.9)
Multiple-substance	2,081 (13.4)	3,305 (32.5)	6,760 (43.4)	9,018 (45.7)	8,970 (47.2)	8,021 (44.3)	6,639 (32.1)	1,139	45,933 (37.1)
**Reason for exposure**									
Unintentional	15,524 (99.8)	2,549 (25.2)	1,879 (12.2)	2,741 (14.1)	3,499 (18.7)	5,185 (29.2)	13,044 (64.3)	2,552	46,973 (38.5)
Unintentional–general	14,946 (96.1)	554 (5.5)	328 (2.1)	385 (2.0)	427 (2.3)	450 (2.5)	865 (4.3)	342	18,297 (15.0)
Unintentional–therapeutic error	548 (3.5)	1,917 (19.0)	1,447 (9.4)	2,193 (11.3)	2,939 (15.7)	4,580 (25.8)	11,947 (58.9)	2,139	27,710 (22.7)
Unintentional–other^b^	27 (0.2)	64 (0.6)	88 (0.6)	115 (0.6)	99 (0.5)	116 (0.7)	185 (0.9)	55	749 (0.6)
Unintentional–unknown	3 (0.0)	14 (0.1)	16 (0.1)	48 (0.2)	34 (0.2)	39 (0.2)	47 (0.2)	16	217 (0.2)
Intentional	2 (0.0)	7,430 (73.9)	13,143 (85.7)	16,263 (83.9)	14,730 (78.9)	11,920 (67.1)	6,020 (29.7)	1,943	71,451 (58.5)
Intentional–suspected suicide	2 (0.0)	5,854 (58.2)	9,867 (64.3)	12,522 (64.6)	11,942 (63.9)	9,527 (53.7)	4,240 (20.9)	1,251	55,205 (45.2)
Intentional–misuse	0 (0.0)	418 (4.2)	1,128 (7.4)	1,412 (7.3)	1,191 (6.4)	1,140 (6.4)	1,120 (5.5)	227	6,636 (5.4)
Intentional–abuse	0 (0.0)	844 (8.4)	1,495 (9.7)	1,459 (7.5)	855 (4.6)	576 (3.2)	276 (1.4)	195	5,700 (4.7)
Intentional–unknown	0 (0.0)	314 (3.1)	653 (4.3)	870 (4.5)	742 (4.0)	677 (3.8)	384 (1.9)	270	3,910 (3.2)
Other^c^	22 (0.1)	86 (0.9)	320 (2.1)	388 (2.0)	445 (2.4)	627 (3.5)	1,220 (6.0)	584	3,692 (3.0)
Unknown	31	91	256	359	348	374	404	182	2,045
**Management site**									
Managed on site (not in a healthcare facility)	10,414 (67.4)	2,107 (20.9)	1,724 (11.1)	2,361 (12.0)	3,049 (16.1)	4,542 (25.2)	11,622 (56.7)	2,787	38,606 (31.3)
Individual already in (en route to) a healthcare facility	3,475 (22.5)	6,979 (69.1)	12,325 (79.4)	15,771 (80.2)	14,641 (77.3)	12,360 (68.6)	7,474 (36.5)	688	73,713 (60.0)
Individual referred by poison center to a healthcare facility	1,509 (9.8)	915 (9.1)	1,371 (8.8)	1,407 (7.2)	1,145 (6.0)	1,002 (5.6)	1,152 (5.6)	1,477	9,978 (8.1)
Other	56 (0.4)	93 (0.9)	108 (0.7)	122 (0.6)	109 (0.6)	126 (0.7)	251 (1.2)	108	973 (0.8)
Unknown	125	62	70	90	78	76	189	201	891
**Highest level of health care received**									
No healthcare facility treatment received^d^	10,470 (69.9)	2,200 (23.0)	1,832 (12.8)	2,483 (13.5)	3,158 (17.6)	4,668 (27.1)	11,873 (60.1)	2,895	39,579 (34.0)
Treated/evaluated and released	3,950 (26.4)	3,155 (33.0)	4,795 (33.5)	5,450 (29.6)	4,679 (26.0)	3,866 (22.5)	2,894 (14.7)	257	29,046 (25.1)
Admitted	352 (2.4)	4,037 (39.3)	7,349 (51.3)	10,092 (55.0)	9,830 (54.8)	8,431 (49.0)	4,655 (23.6)	221	44,967 (39.0)
Admitted to a critical care unit	144 (1.0)	724 (7.6)	1,949 (13.6)	3,136 (17.1)	3,178 (17.7)	2,750 (16.0)	1,546 (7.8)	60	13,487 (11.7)
Admitted to a non-critical care unit	204 (1.4)	763 (8.0)	1,466 (10.2)	2,218 (12.1)	2,132 (11.9)	2,169 (12.6)	1,709 (8.7)	51	10,712 (9.3)
Admitted to a psychiatric facility	4 (0.0)	2,550 (26.7)	3,934 (27.5)	4,738 (25.8)	4,520 (25.2)	3,512 (20.4)	1,400 (7.1)	110	20,768 (18.0)
Refused referral/did not arrive at healthcare facility	204 (1.4)	162 (1.7)	353 (2.5)	363 (2.0)	297 (1.7)	252 (1.5)	326 (1.7)	420	2,377 (2.0)
Unknown^e^	603	602	1,269	1,363	1,058	889	940	1,468	8,192
**Medical outcome**									
No effect	7,410 (49.6)	2,675 (27.7)	3,217 (22.2)	3,731 (20.1)	3,502 (19.4)	3,139 (18.1)	3,462 (17.4)	421	27,557 (23.6)
Minor effect	1,129 (7.6)	3,389 (35.1)	5,331 (36.8)	6,473 (34.9)	6,065 (33.6)	5,215 (30.1)	3,867 (19.4)	468	31,937 (27.4)
Serious medical outcome	179 (1.2)	1,729 (17.9)	4,010 (27.6)	5,883 (31.7)	5,583 (30.9)	4,935 (28.4)	3,147 (15.8)	193	25,659 (22.1)
Moderate effect	167 (1.1)	1,536 (15.9)	3,373 (23.3)	4,891 (26.4)	4,601 (25.5)	4,065 (23.4)	2,589 (13.0)	167	21,389 (18.3)
Major effect	12 (0.1)	191 (2.0)	630 (4.3)	972 (5.2)	964 (5.3)	845 (4.9)	537 (2.7)	23	4,174 (3.6)
Death	0 (0.0)	2 (0.0)	7 (0.0)	20 (0.1)	18 (0.1)	25 (0.1)	21 (0.1)	3	96 (0.1)
Not followed^f^	6,219 (42.1)	1,851 (19.4)	1,930 (13.8)	2,469 (12.9)	2,905 (16.1)	4,050 (23.7)	9,417 (47.2)	2,579	31,420 (27.0)
Unknown^g^	642	512	1,110	1,195	967	767	795	1,600	7,588
**Total (row %)**	**15**,**579 (12.6)**	**10**,**156 (8.2)**	**15**,**598 (12.6)**	**19**,**751 (16.0)**	**19**,**022 (15.4)**	**18**,**106 (14.7)**	**20**,**688 (16.8)**	**5**,**261**	**124**,**161 (100.0)**

^a^Column percentages may not add to 100.0% due to rounding error

^b^ Includes unintentional-environmental, unintentional-occupational, and unintentional-misuse

^c^Includes other-contamination/tampering, other-malicious, other-withdrawal, adverse reaction-drug or other

^d^ Includes cases for which management site was recorded “managed on site (not in a healthcare facility)” or “other”

^e^ Includes patient lost to follow-up/ left against medical advice/unknown

^f^ Includes “not followed (minimal clinical effects possible)” and “not followed (judged as a non-toxic exposure)”

^g^ Includes “unable to follow (judged as a potentially toxic exposure)”

**Table 2:** Characteristics of exposures involving gabapentin or pregabalin as the primary substance reported to the national poison data system by bubstance, 2012–2022

Characteristics	Substance		
	Gabapentin *n* (%)^a^	Pregabalin *n* (%)^a^	Total *n* (%)^a^
**Sex**			
Male	43,525 (41.0)	6,741 (38.6)	50,266 (40.6)
Female	62,731 (59.0)	10,720 (61.4)	73,451 (59.4)
Unknown	383	61	444
**Age group**			
≤5 years	12,750 (12.5)	2,829 (16.9)	15,579 (13.1)
6-19 years	8,892 (8.7)	1,264 (7.5)	10,156 (18.5)
20-29 years	14,038 (13.7)	1,560 (9.3)	15,598 (13.1)
30-39 years	17,385 (17.0)	2,366 (14.1)	19,751 (16.6)
40-49 years	16,322 (16.0)	2,700 (16.1)	19,022 (16.0)
50-59 years	15,340 (15.0)	2,766 (16.5)	18,106 (15.2)
≥60 years	17,411 (17.1)	3,277 (19.6)	20,688 (17.4)
Unknown	4,501	760	5,261
**Type of exposure**			
Single-substance	66,819 (62.7)	11,409 (65.1)	77,228 (63.0)
Multiple-substance	39,820 (37.3)	6,113 (34.9)	45,933 (37.0)
**Reason for exposure**			
Unintentional	39,480 (37.6)	7,493 (43.5)	46,973 (38.5)
Unintentional–general	15,136 (14.4)	3,161 (18.4)	18,297 (15.0)
Unintentional–therapeutic error	23,527 (22.4)	4,183 (24.3)	27,710 (22.7)
Unintentional–other^b^	642 (0.6)	107 (0.6)	749 (0.6)
Unintentional–unknown	175 (0.2)	42 (0.2)	217 (0.2)
Intentional	62,498 (59.6)	8,953 (52.1)	70,451 (58.5)
Intentional–suspected suicide	48,701 (46.4)	6,504 (37.8)	54,205 (45.2)
Intentional–misuse	5,617 (5.4)	1,019 (5.9)	6,636 (5.4)
Intentional–abuse	4,848 (4.6)	852 (5.0)	5,700 (4.7)
Intentional–unknown	3,332 (3.2)	578 (3.4)	3,910 (3.2)
Other^c^	2,939 (2.8)	753 (4.4)	3,692 (3.0)
Unknown	1,722	323	2,045
**Management site**			
Managed on site (not in a healthcare facility)	32,887 (31.1)	5,719 (32.9)	38,606 (31.3)
Individual already in (en route to) a healthcare facility	63,709 (60.2)	10,004 (57.5)	73,713 (59.8)
Individual referred by poison center to a healthcare facility	8,466 (8.0)	1,512 (8.7)	9,978 (8.1)
Other	822 (0.8)	151 (0.9)	973 (0.8)
Unknown	755	136	891
**Highest level of health care received**			
No healthcare facility treatment received^d^	33,709 (33.9)	5,870 (35.8)	39,579 (34.1)
Treated/evaluated and released	24,916 (25.0)	4,130 (25.2)	29,046 (25.0)
Admitted	38,954 (39.1)	6,033 (36.7)	44,967 (38.7)
Admitted to a critical care unit	11,242 (11.3)	2,245 (13.7)	13,487 (11.6)
Admitted to a non-critical care unit	9,129 (9.2)	1,583 (9.6)	10,712 (9.2)
Admitted to a psychiatric facility	18,583 (18.6)	2,205 (13.4)	20,768 (17.9)
Refused referral/did not arrive at healthcare facility	1,996 (2.0)	381 (2.3)	2,377 (2.0)
Unknown^e^	7,084	1,108	8,192
**Medical outcome**			
No effect	23,874 (23.8)	3,683 (22.4)	27,557 (23.6)
Minor effect	27,631 (27.6)	4,306 (26.2)	31,937 (27.4)
Serious medical outcome	21,505 (21.4)	4,154 (25.2)	25,659 (22.0)
Moderate effect	17,964 (17.9)	3,425 (20.8)	21,389 (18.3)
Major effect	3,452 (3.4)	722 (4.4)	4,174 (3.6)
Death	89 (0.1)	7 (0.0)	96 (0.1)
Not followed^f^	27,135 (27.1)	4,285 (26.1)	31,420 (26.9)
Unknown^g^	6,494	1,094	7,588
**Total (row %)**	**106**,**639 (85.9)**	**17**,**522 (14.1)**	**124**,**161 (100.0)**

^a^Column percentages may not add to 100.0% due to rounding error

^b^Includes unintentional-environmental, unintentional-occupational, and unintentional-misuse

^c^Includes other-contamination/tampering, other-malicious, other-withdrawal, adverse reaction-drug or other

^d^Includes cases for which management site was recorded “managed on site (not in a healthcare facility)” or “other”

^e^Includes patient lost to follow-up/ left against medical advice/ unknown

^f^Includes “not followed (minimal clinical effects possible)” and “not followed (judged as a non-toxic exposure)”

^g^Includes “unable to follow (judged as a potentially toxic exposure)”

## Reason for exposure

Suspected suicide was the most common (45.2%) reason for exposure involving gabapentin or pregabalin as the primary substance for all individuals combined (Table 1). However, the reason for exposure varied among age groups. Unintentional-general was the most common reason (96.1%) among ≤ 5-year-olds, therapeutic errors accounted for most (58.9%) exposures among individuals ≥ 60 years old, and suspected suicides represented most exposures among the other age groups.

Among the 55,205 suspected suicide exposures, 28.0% were associated with a serious medical outcome, including 53 fatalities. Exposures related to suspected suicides were more likely to be associated with a serious medical outcome (RR: 2.63, 95% CI: 2.57–2.70) than exposures related to all other reasons for exposure combined. Suspected suicides were also more likely to be associated with admission to a CCU or non-CCU (RR: 3.07, 95% CI: 3.00-3.15) than all other reasons for exposure combined.

## Single-substance and multiple-substance exposures

Most (62.9%) exposures involved a single substance, which was especially true among children ≤ 5 years old (86.6%) (Table 1). Single-substance exposures accounted for more than three-fourths (78.6%) of unintentional exposures, including 83.5% of unintentional-general exposures and 75.7% of therapeutic errors. Single-substance exposures were less predominant (52.2%) among intentional exposures, including intentional-misuse (69.8%), intentional-abuse (55.6%), and suspected suicide (49.5%). Multiple-substance exposures with gabapentin or pregabalin as the primary substance were more likely to be associated with a serious medical outcome (RR: 2.53, 95% CI: 2.47–2.58) or admission to a CCU or non-CCU (RR: 2.40, 95% CI: 2.35–2.46) than single-substance exposures. Among these 45,933 multiple-substance exposures, the top five most commonly involved substances (in addition to gabapentin or pregabalin, which were the primary substances) were (1) ethanol (alcoholic beverages) (25.9%), (2) benzodiazepines (18.2%), (3) atypical antipsychotics (7.5%), (4) trazodone (4.5%), and (5) ibuprofen (3.2%).

### Highest level of health care received

Although most exposures reported to a US PC did not receive care at a healthcare facility (34.0%) or were treated/evaluated and released (25.1%); 11.7% were admitted to a CCU and 9.3% were admitted to a non-CCU (Table 1). Individuals ≤ 5 years old or ≥ 60 years old were less likely to be admitted to a CCU or non-CCU (RR: 0.39, 95% CI: 0.38–0.41) than the other age groups combined. Individuals exposed to gabapentin were less likely to be admitted to a CCU or non-CCU (RR: 0.88, 95% CI: 0.85–0.90) and more likely to be admitted to a psychiatric facility (RR: 1.39, 95% CI: 1.33–1.45) than individuals exposed to pregabalin.

### Medical outcome

Most reported exposures were associated with a minor effect (27.4%) or no effect (34.0%), while 22.1% experienced a serious medical outcome. The age group with the greatest proportion of serious medical outcomes was 30-39-year-olds (31.7%), followed by individuals 40–49 years old (30.9%) and 50–59 years old (28.4%) (Table 1). Individuals ≤ 5 years old or ≥ 60 years old were less likely to experience a serious medical outcome than individuals in the other age groups combined (RR: 0.34, 95% CI: 0.33–0.35). Individuals exposed to gabapentin were less likely to experience a serious medical outcome than those exposed to pregabalin (RR: 0.85, 95% CI: 0.83–0.87).

There were 96 fatalities reported during the study period, including 25 among individuals 50–59 years old, followed by individuals ≥ 60 years old (*n* = 21), 30–39 years old (*n* = 20), and 40–49 years old (*n* = 18). Fifty-two decedents were female, 58 were associated with multiple-substance exposures, 53 were identified as suspected suicide, and 89 involved gabapentin as the primary substance (including 36 as the single-substance). Among the 81 fatalities that had a relative contribution to fatality determination by America’s Poison Centers, gabapentin or pregabalin was undoubtedly responsible for the death in 11 (13.6%), probably responsible in 37 (45.7%), contributory in 17 (21.0%), and unknown in 16 (19.8%).

### Trends

**Fig. 1:**
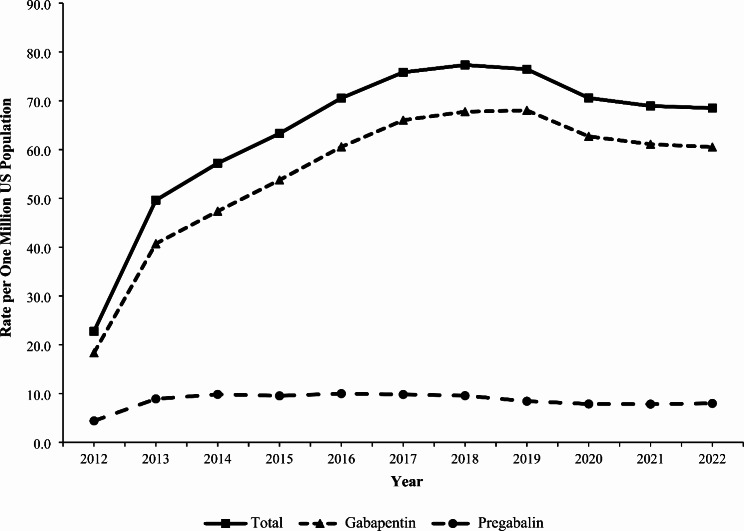
Annual Rate of Gabapentin and Pregabalin Exposures Reported to the National Poison Data System by Substance, 2012–2022

**Fig. 2:**
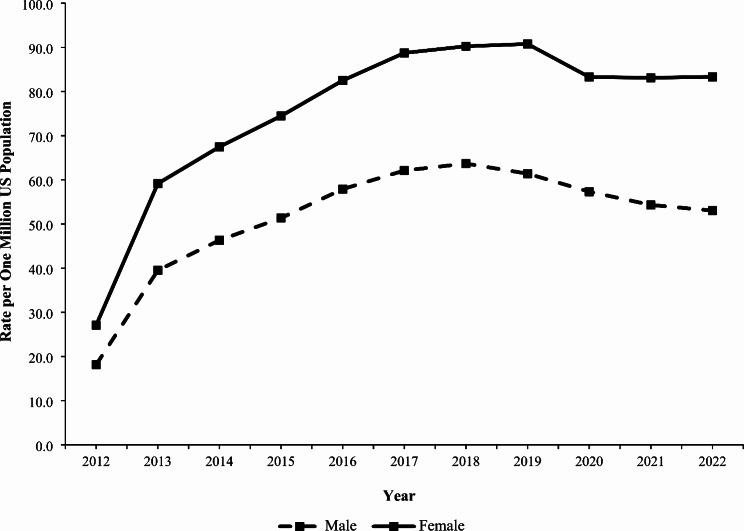
Annual Rate of Gabapentin and Pregabalin Exposures Reported to the National Poison Data System by Sex, 2012–2022

**Fig. 3:**
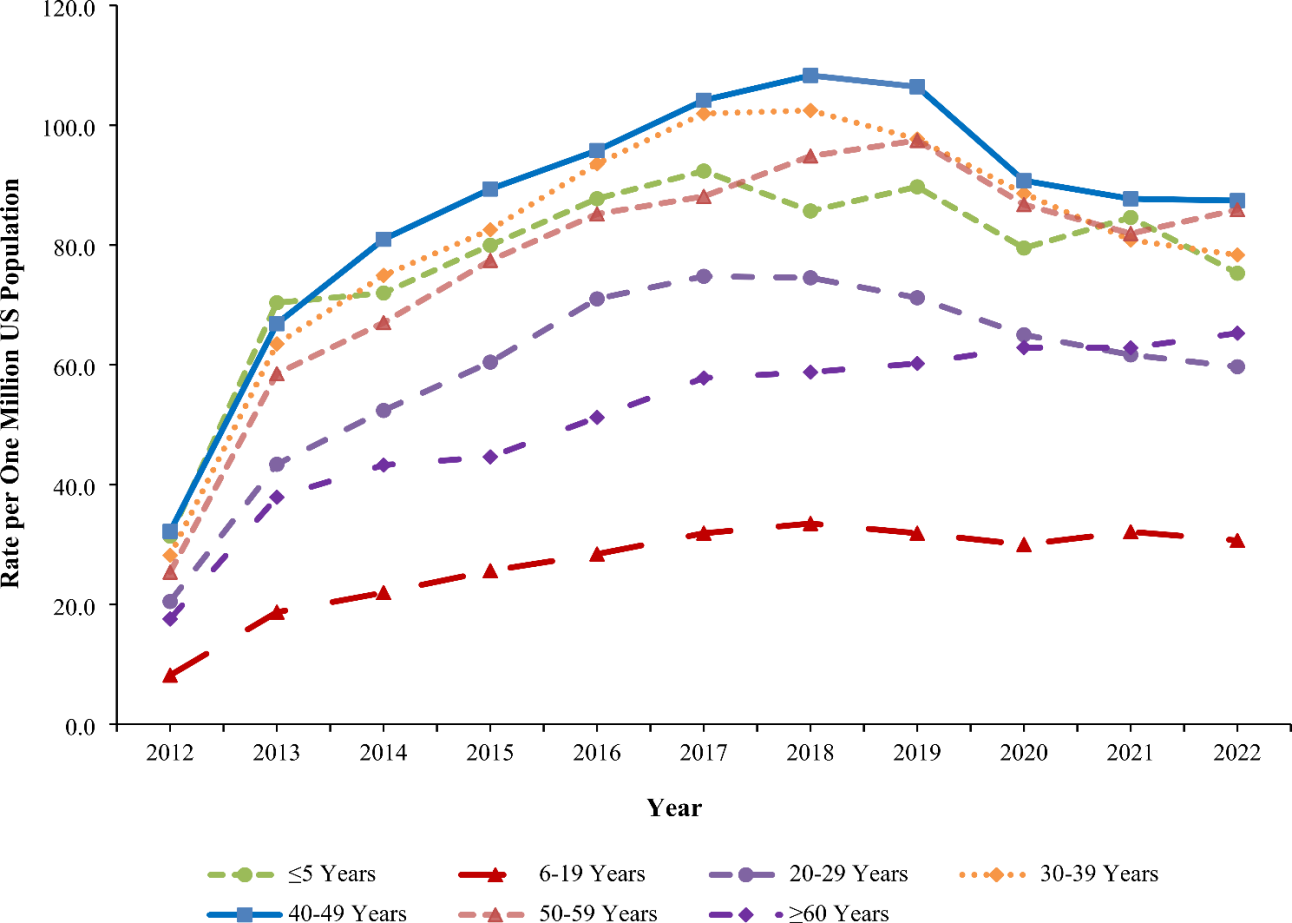
Annual Rate of Gabapentin and Pregabalin Exposures Reported to the National Poison Data System by Age Group, 2012–2022

**Fig. 4:**
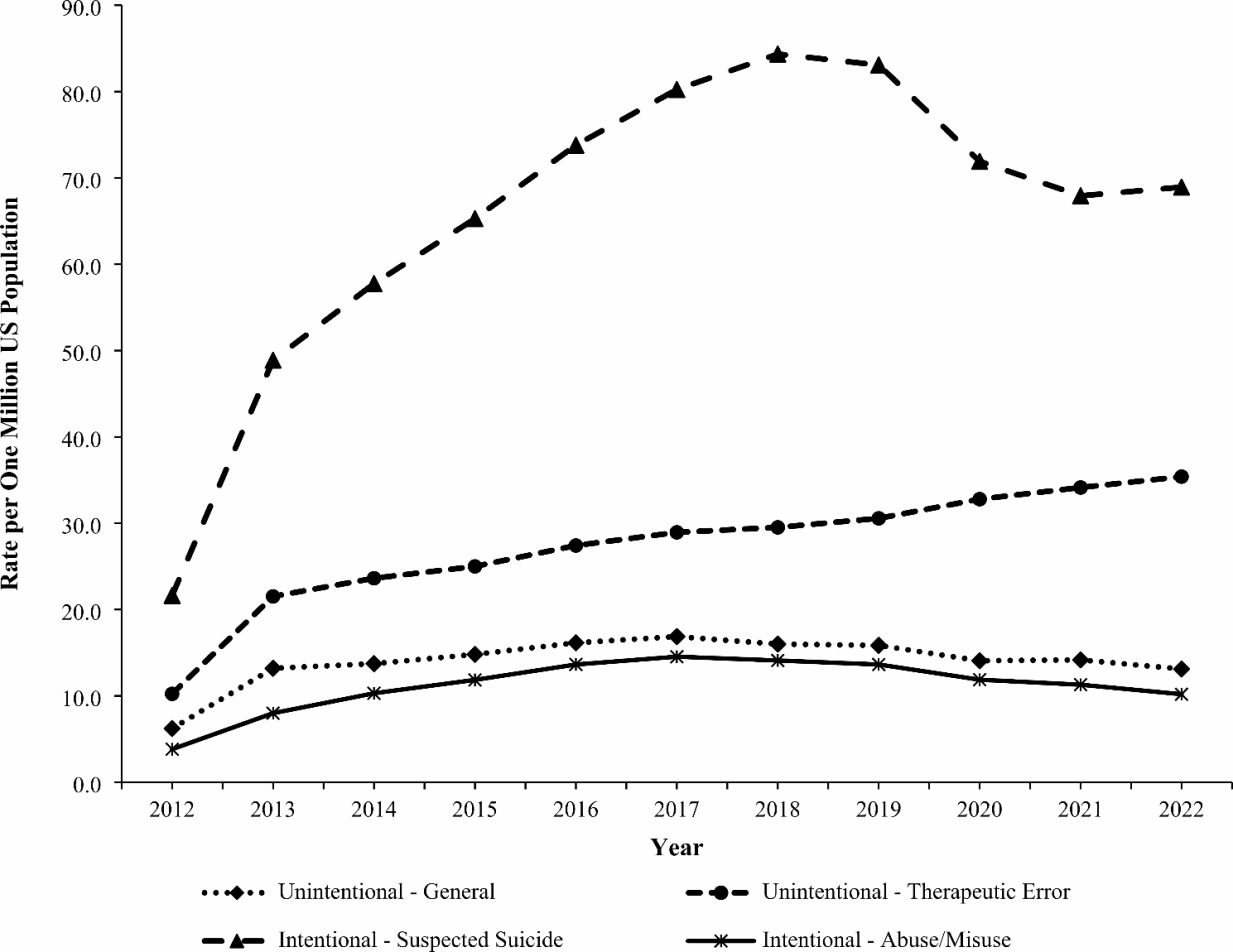
Annual Rate of Gabapentin and Pregabalin Exposures Reported to the National Poison Data System by Selected Reason for Exposure, 2012–2022

The rate of exposure per one million US population involving gabapentin or pregabalin (as either the primary or non-primary substance) reported to US PCs increased by 236.1% from 22.7 in 2012 to 76.5 in 2019 (*P* < 0.001), followed by a non-significant decrease to 68.5 in 2022 (*P* = 0.068) (Fig. 1). The rate of gabapentin exposures alone increased 270.8% from 18.3 in 2012 to 68.0 in 2019 (*P* < 0.001), followed by a decrease to 60.5 in 2022 (*P* = 0.052). In contrast, the rate of pregabalin exposures did not change significantly during the study period (*P* = 0.782) (Fig. 1). The rate trend for gabapentin and pregabalin exposures was similar for females and males, except males experienced a 13.6% decline from 2019 to 2022 (*P* = 0.038). Females experienced a higher rate than males throughout the study period, with a growing difference between the rates observed over time (Fig. 2). There was variation of the rate trends among age groups, with the highest rates observed among 40-49-year-olds and 30-39-year-olds (Fig. 3). Although the rates of intentional misuse or abuse and unintentional-general exposures remained relatively unchanged, the rate of therapeutic errors increased consistently during the study period (*P* < 0.001). The rate of suspected suicide increased by 290.1% from 21.6 in 2012 to 84.4 in 2018 (*P* < 0.001) and then decreased by 18.3% to 68.9 in 2022 (*P* = 0.007) (Fig. 4).

**Fig. 5:**
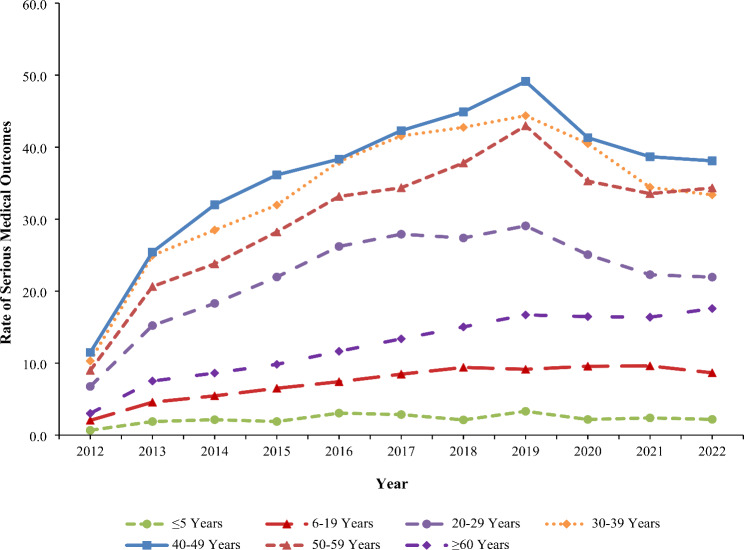
Annual Rate of Serious Medical Outcomes per One Million US Population Associated with Gabapentin and Pregabalin Exposures Reported to the National Poison Data System by Age Group, 2012–2022

The rate trends of serious medical outcomes varied by age group. The rate remained constant among children ≤ 5 years old (*P* = 0.119) but increased significantly among 6-19-year-olds (*P* < 0.001) and ≥ 60-year-olds (*P* < 0.001) from 2012 to 2022 (Fig. 5). For the other age groups (i.e., individuals 20–29, 30–39, 40–49, and 50–59 years old), the rate of a serious medical outcome increased significantly from 2012 to 2019, followed by a significant decrease from 2019 to 2022 (*P*-values < 0.05) (Fig. 5).

## Discussion

During the 11-year study period, US PCs received 124,161 reports of gabapentin or pregabalin exposure as the primary substance, which averaged 6,208 exposures annually. There was a female predominance, which is consistent with findings from previous studies (Faryar et al. 2019; Reynolds et al. 2020) Suspected suicide was the most common reason for exposure in this study, accounting for 45% of exposures, which aligns with previous research (Faryar et al. 2019; Klein-Schwartz et al. 2003; Reynolds et al. 2020; Toce et al. 2022). Suspected suicides represented more than half of exposures among individuals 6–59 years old. The trend for the rate of suspected suicide was a driver of the trend observed for gabapentin and pregabalin exposures during the study period. Recommendations to help prevent these suicides include prescriber education, increased screening for co-occurring substance use disorders, mood disorders, and suicidal ideation, and judicious prescribing practices for gabapentin and pregabalin (Farah et al. 2023).

Approximately 10% of gabapentin and pregabalin exposures in this study were attributed to intentional misuse or abuse. Previous reports have identified the intentional misuse or abuse of gabapentin and pregabalin and associated consequences (Tharp et al. 2019; Mattson et al. 2022; Goins et al. 2021; Peckham et al. 2018a, 2018b; Hagg et al. 2020; Schifano 2014). In particular, the concurrent use of opioids and gabapentinoids has been linked to an increased likelihood of hospitalization and opioid-related fatalities, which prompted an update to the Beers criteria by the American Geriatric Society, advising caution against dual therapy among older adults (Goins et al. 2021; Peckham et al. 2018a; American American Geriatrics Society 2019; Gomes et al. 2017). Although pregabalin is a federally controlled substance, gabapentin is not, which has led to calls for the US Drug Enforcement Administration to schedule gabapentin the same as pregabalin (Peckham et al. 2018a, 2018b). Gabapentin is a scheduled drug in some states and legislation has been introduced in a number of other states to reclassify or monitor its use (Sharkey 2023; Peckham et al. 2018b;  Throckmorton et al. 2018).

The rate of exposures reported to US PCs involving gabapentin and pregabalin increased from 2012 to 2019 and then decreased slightly from 2019 to 2022. This trend generally parallels the number of gabapentin prescriptions dispensed nationally, which increased by 70.4% from 2011 to 2015, followed by a slower increase of 20.0% from 2015 to 2019 and then 3.4% from 2019 to 2021 (US Drug Enforcement Administration 2023). The reasons for the observed trend are likely multifactorial and cannot be determined by this study. Potential factors may include a 2019 drug safety communication by the FDA that required warnings about respiratory depression be added to the prescribing information for gabapentinoids (US Food and Drug Administration 2020), scheduling of gabapentin as a controlled substance in a number of states (Sharkey 2023), the US opioid crisis (Spencer et al. 2002), and the COVID-19 pandemic. The rate of suspected suicide was a driver of the observed overall rate trend for gabapentin and pregabalin exposures, and it was similar to that for suicide-related fatalities (from all causes) in the US, which increased from 2001 to 2018, declined in 2019 and 2020, before increasing again in 2021 (Garnett and Curtin 2001).

In this study, pregabalin was more likely than gabapentin to be associated with a serious medical outcome or admission to a CCU or non-CCU, which may be attributable to multiple factors, including pregabalin’s higher potency, quicker rate of absorption, and greater bioavailability compared with gabapentin (Schifano 2014). Although statistically significant, the differences between the two medications were not large and should be interpreted with caution, especially given that the dosage of gabapentin versus that of pregabalin was not accounted for in this study, not all exposures were captured by the NPDS, and not all captured exposures were followed to a known outcome. Regardless, because gabapentin accounted for 86% of exposures in this study, its outcomes were important in terms of comparative frequency.

The reason for exposure among children ≤ 5 years old was usually “unintentional-general,” which represents exploratory behaviors. As young children gain mobility, they explore their environment without recognizing potential danger. Most medical outcomes in this age group were minor or no effects; however, approximately 1% were associated with a serious medical outcome. Prevention strategies for these incidents include (1) storing these medications out of reach and sight of children, preferably in a locked location, (2) using child-resistant containers, and (3) having the poison helpline number (800-222-1222) available in case an exposure does occur (US Centers for Disease Control and Prevention 2023).

Among individuals ≥ 60 years old, therapeutic errors were the most predominant reason for exposure. The likelihood of these errors occurring is exacerbated by the greater number of prescription medications used by older adults (National Institute on Drug Abuse 2020). The use of pill organizers may help prevent common errors; however, these should be child-resistant to mitigate unintended child exposures. The use of medication reminders and tracking systems, and when necessary, having a second adult regularly conduct medication reconciliation or assist in medication administration to older adults, may be helpful (Lavan et al. 2016).

Careful consideration is advised when prescribing gabapentinoids, especially to high-risk individuals, such as persons using opioids, persons with substance use disorders, older adults, and those susceptible to respiratory depression (US Food and Drug Administration 2020; Hagg et al. 2020; Chincholkar et al. 2020). To mitigate the possibility of adverse effects, clinicians should closely monitor patient response, and patients should promptly report any side effects (Chincholkar et al. 2020). The risk for intentional misuse and abuse should also be monitored by the prescribing clinician and other healthcare personnel and family members involved in the patient’s care (Hagg et al. 2020; Chincholkar et al. 2020).

### Study limitations

This study has several limitations. Because the NPDS is a passive surveillance system, this study underestimates the incidence of gabapentin and pregabalin exposures. Exposures may be treated in emergency departments, urgent care facilities, or other healthcare settings without a PC being contacted, and some exposures may not receive medical care at all. The proportion of all true exposures captured by the NPDS may change over time. There also may be bias regarding which cases are reported to a PC, based on an individual’s age, severity of the exposure, or other factors (Hoffman 2007). Data are self-reported and cannot be completely verified by the PCs or America’s Poison Centers. Cases reported to the NPDS do not necessarily represent a poisoning or overdose. Repeat exposures involving the same individual were not identifiable because we did not have access to personal identifiers. Medication dose was not analyzed in this study. Although NPDS protocols for quality control and follow-up are used, data errors and missing data may occur. Despite these limitations, the NPDS provides a large national database useful for investigating the characteristics and trends of gabapentin and pregabalin exposures in the US.

## Conclusions

The rate of gabapentin and pregabalin exposures reported to US PCs increased by more than 230% from 2012 to 2019 before plateauing from 2019 to 2022. The observed rate trend was driven primarily by gabapentin exposures and by cases associated with suspected suicide. Although most exposures were associated with a minor or no effect, 22% of individuals experienced a serious medical outcome, including 96 fatalities. These findings contribute to the discussion of rescheduling gabapentin as a federally controlled substance, which is the current status of pregabalin. Prevention of suicide associated with gabapentin and pregabalin merits special attention.

## Data Availability

Data analyzed in this study were from the National Poison Data System, which is a proprietary database owned and managed by America’s Poison Centers. Data requests should be submitted to America’s Poison Centers at: https://www.aapcc.org/national-poison-data-system.

## Declarations

### Disclaimer by America’s Poison Centers

America’s Poison Centers maintains the National Poison Data System (NPDS), which houses de-identified case records of self-reported information collected from callers during exposure management and poison information calls managed by the country’s poison control centers (PCCs). NPDS data do not reflect the entire universe of exposures to a particular substance as additional exposures may go unreported to PCCs; accordingly, NPDS data should not be construed to represent the complete incidence of US exposures to any substance(s). Exposures do not necessarily represent a poisoning or overdose, and America’s Poison Centers is not able to completely verify the accuracy of every report. Findings based on NPDS data do not necessarily reflect the opinions of America’s Poison Centers.

## Abbreviations

CCU: Critical Care Unit
CI: Confidence Interval
FDA: Food and Drug Administration
NPDS: National Poison Data System
PC: Poison Center
RR: Risk Ratio
US: United States
